# Knockdown of glucose-regulated protein 78 decreases the invasion, metalloproteinase expression and ECM degradation in hepatocellular carcinoma cells

**DOI:** 10.1186/1756-9966-31-39

**Published:** 2012-04-30

**Authors:** Hongdan Li, Huijuan Song, Junsheng Luo, Jia Liang, Song Zhao, Rongjian Su

**Affiliations:** 1 Key Lab of Molecular and Cellular Biology of the Education Department of Liaoning Province, Central Laboratory of Liaoning Medical College, Songpo Road, Jinzhou, Liaoning, People’s Republic of China; 2 The First Affiliated Hospital of Liaoning Medical College, Jinzhou, Liaoning, People’s Republic of China

**Keywords:** GRP78, ECM degradation, MMP-2, JNK

## Abstract

**Background:**

We have reported previously that overexpression of glucose-regulated protein 78 (GRP78) promotes the invasion of hepatocellular carcinoma. However, whether GRP78 knockdown affects the extracellular matrix degradation has not been elucidated. Here we are going to determine whether GRP78 knockdown affect the ECM degradation and the role of MMP-2 and MMP-9 in these process in hepatocellular carcinoma cells.

**Methods:**

Human hepatocellular carcinoma cell line SMMC7721 and HepG2 were cultured in DMEM supplemented with 10% FBS, RT-PCR and western blot were used to detect the endogenous expression of GRP78, MMP-2, MMP-9 and TIMP-2 in SMMC7721 and HepG2. GRP78 shRNAs were transfected using lipofection2000. Transwell assay and wound healing assay were used to analyze the invasion of each transfectant. Gelatin zymography and FITC-gelatin degradation assay were employed to investigate the capabilities of ECM degradation of each transfectant. MTT assay was used to determine the proliferation status. Western blot was employed to detect the expression of matrix metalloproteinase 2(MMP-2), MMP-9, MMP-14, and tissue inhibitor of metalloproteinases 2(TIMP-2), focal adhesion kinase (FAK), ERK1/2, JNK and Src.

**Results:**

According to the expression levels of GRP78, MMP-2, MMP-9, MMP-14 and TIMP-1 in hepatocellular carcinoma cell lines SMMC7721 and hepG2, we used SMMC7721 as the in vitro invasion model for further functional analysis. Using this model, we found that GRP78 knockdown decreased the invasion of tumor cells, and this inhibitory effect was independent of cell proliferation. In hepatocellular carcinoma cells, Grp78 knockdown inhibited ECM degradation and the decreased activity and expression of MMP-2, but not MMP-9 contributed largely to this impact. Further analysis revealed that the decreased activity and expression of MMP-2 is mediated by JNK.

**Conclusion:**

Knockdown of GRP78 decreases ECM degradation, and downregulates the expression and activity of MMP-2 and TIMP-2. These results further demonstrate that GRP78 is a potential target for inhibiting the invasion of hepatocellular carcinoma cells.

## Background

Hepatocellular carcinoma (HCC), also called hepatoma, is the most frequent type of primary liver cancer and one of the leading causes of cancer death worldwide, which caused over 600,000 deaths per year [1]. Invasion and metastasis are the most critical reason for the poor prognosis of HCC patients [2].

Glucose-regulated protein 78(GRP78) is present at a basal level in normal tissues. However it is overexpressed in almost all the human cancers and plays important role in anti-apoptotic process of cancer cells [3]. GRP78, which has been regarded as a endoplasmic reticulum(ER) chaperone previously, is a multifunctional protein [4,5]. Recently, lots of data have demonstrated that Grp78 is involved in the regulation of invasion and metastasis of many human cancers including breast, prostate, gastric, lung, liver cancers [6-10]. Although we have reported that GRP78 facilitates the invasion of hepatocellular carcinoma cells, whether GRP78 plays a role in ECM degradation is still not determined.

The invasion and metastasis of cancer cells is a complex process which is mainly determined by the following events: (1) extracellular matrix (ECM) degradation, (2) the arrangement of cytoskeleton, (3) cell polarity formation [11-13]. These processes are tightly regulated by temporally and spatially regulated expression and activation of many signal molecules including focal adhesion kinase (FAK), Src, c-Jun N-terminal kinase (JNK) [14,15].

Matrix metalloproteinases (MMPs) are a family of related zinc-dependent proteinases that degrade most extracellular matrix [16]. So far, nearly 20 members of the MMP family that share common structural and functional elements have been identified [17]. Among them, MMP-2 and MMP-9 are the most concerned and their functions have been well-characterized. They are believed to play important role in the invasive process and high level expression or activation of MMPs is associated with the invasion and metastasis of cancer cells [18]. The activity of MMP-2 and MMP-9 is regulated by many factors. Recent studies have revealed that the membrane type metalloproteinases (MT-MMP) and the tissue inhibitor of metalloproteinases (TIMP) play coordinately in the regulation of MMPs activity. MMP-2 is activated by complexing with MT-MMP1 (MMP14) and TIMP-2. However, MMP-9 is activated by binding with TIMP-1 [19-21].

In this article, we knockdown GRP78 level in hepatocellular carcinoma cell line SMMC7721, and explored the effect of Grp78 knockdown on the ECM degradation and the underlying mechanism.

## Results

### Endogenous expression of GRP78 in hepatocellular carcinoma cells SMMC7721 and HepG2

**Figure 1:**
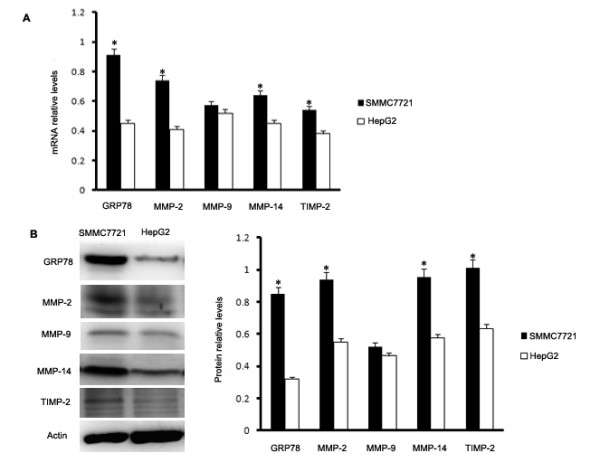
**Endogenous expression of GRP78 in hepatocellular carcinoma cells. (A)** Quantative RT-PCR analysis for mRNA levels of GRP78, MMP-2, MMP-9, MMP-14, and TIMP-2 in hepatocellular carcinoma cell lines SMMC7721 and HepG2. The mRNA contents in the cells were presented as the relative levels normalized to 18 S mRNA. **(B)** Western blot analysis for protein levels of GRP78, MMP-2, MMP-9, MMP-14, and TIMP-2 in hepatocellular carcinoma cell lines SMMC7721 and HepG2. Protein levels were expressed as the ratio of target protein over β-actin. All the experiments were repeated for three times, the values were presented as $\bar{x}$± SE and analyzed by the students’ *t*-test. (Columns,mean of three separate experiments; bars, SE; *, values significantly different at the 5% levels).

To investigate the expression of GRP78 in hepatocellular carcinoma cell lines, we examined GRP78 levels in SMMC7721 and HepG2, which are two kinds of widely used hepatocellular carcinoma cell lines, using quantitative RT-PCR and western blot and the data were analyzed by the students’ *t* test. The results revealed that GRP78 was expressed in both SMMC7721 and HepG2 although with different levels. GRP78 level in SMMC7721 cells was significantly higher than that in HepG2 cells at both the mRNA level (p = 0.024) and the protein level (p = 0.001) (Figure 1A and B). We also examined the MMP-2, MMP-9, MMP-14 and TIMP-2 levels at mRNA and protein levels. As shown in Figure 1A and B, the MMP-2, MMP-14 and TIMP-2 levels in SMMC7721 cells were significantly higher than in HepG2 cells (p < 0.05 at mRNA level and p < 0.01 at protein level), however, the difference between the expression of MMP-9 in SMMC7721 and HepG2 was not significant at both mRNA level and protein level (p = 0.069).

### Screening the knockdown effect of GRP78-shRNAs and establishment of cell clones that stably expressing shGRP78

**Figure 2:**
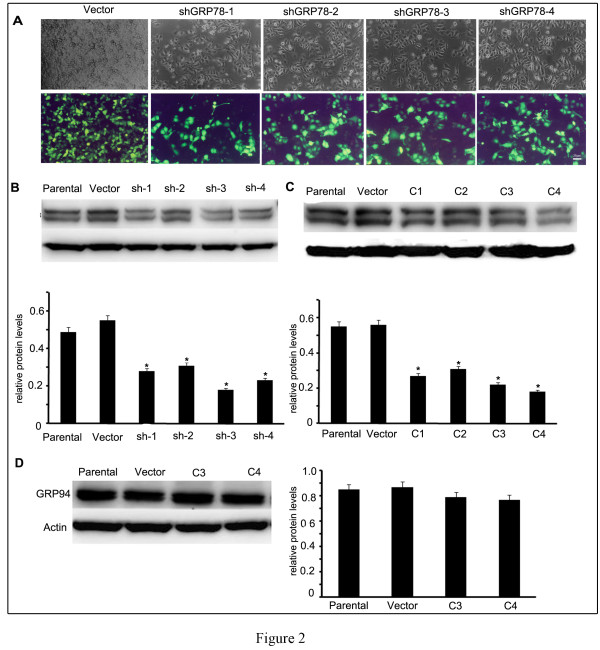
**Screening of the effect of GRP78-shRNAs and the establishment of cell clones that stably expressing GRP78-shRNA. (A)** Fluorescence observation of the transfection efficiencies of shGRP78s in SMMC7721 cells. ShGRP78s containing GFP tag were introduced into SMMC7721 cells as described under “materials and methods”. After 72 h, GFP fluorescence were observed by inverted fluorescent microscope(scale bar:25 μm). **(B)** Western blot analysis of GRP78 levels in GRP78-shRNAs transiently transfected cells. The GRP78 levels were presented as the ratio of GRP78 to β-actin. **(C)** Western blot analysis of GRP78 levels in cells that stably expressing shGRP78-3. The contents of GRP78 were expressed as the ratio of GRP78 toβ-actin. **(D)** Western blot analysis of GRP94 levels in clone C3 and C4 that stably expressing shGRP-3. The contents of GRP94 were expressed as the ratio of GRP94 to β-actin. All the experiments were repeated for three times, the values were presented as $\bar{x}$± SE and analyzed by One-Way ANOVA (Columns,mean of three separate experiments; bars, SE; *, values significantly different at the 5% levels).

Based on the expression status of GRP78, MMP-2, MMP-9, MMP-14 and TIMP-2 in hepatocellular carcinoma cell lines SMMC7721 and HepG2, we choose SMMC7721 to establish the in vitro invasion model for further research. To identify the silencing efficiencies of GRP78-shRNAs (abbreviated as shGRP78 below), we transiently transfected each shGRP78 into SMMC7721 cells, blank vector pEGFP-N1 was transfected at the same time as control. Three days after transfection, GFP fluorescence was directly observed with inverted microscope (Figure 2A). The level of GRP78 in each pool was determined by western blot. We found that each shGRP78 downregulated GRP78 expression with varying degrees. The shGRP78-3 downregulated Grp78 level to ~36.3% compared with control cells, however GRP78 levels in other three shGRP78 transfected cells were >50% compared with control cells (Figure 2B). According to these results, we introduced shGRP78-3 into SMMC7721 and screened the cells that expressing GRP78 at a relative low levels. The clones that stably expressing shGRP78-3 were selected by adding G418(400 μg/ml) in the culture medium for 2–3 weeks. Four clones were randomly chosen and the expressions of GRP78 were detected by western blot (Figure 2C). In the 4 chosen clones, GRP78 levels in clone 3 (abbreviated as C3 below) was ~39.5% of that in control cells, the clone 4 (abbreviated as C3 below) was ~32.7% of that in control cells. So we choose C3 and C4 for further functional analysis. To confirm the specificity of shGRP78-3, we detected the expression of GRP94 in C3 and C4. The results revealed that transfection of shGRP78-3 did not affect the expression of GRP94 (Figure 2D).

### GRP78-silencing decreased the invasion and metastasis of SMMC-7721

**Figure 3:**
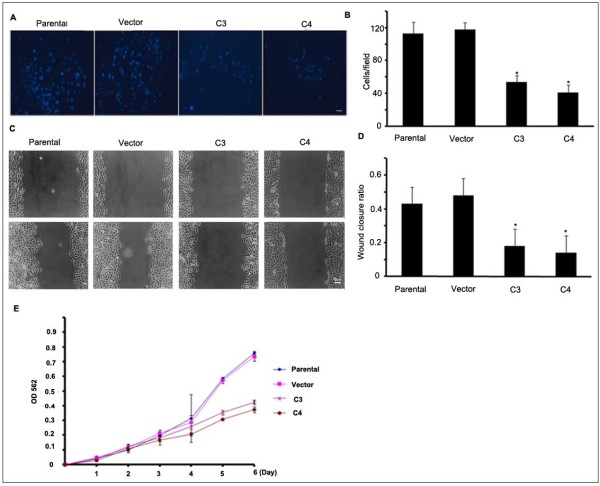
**GRP78 silencing inhibited the invasion and metastasis of SMMC7721. (A)** Transwell analysis of the invasion capability of the cells that stably expressing shGRP78-3. The invaded cells were stained with Hochest33258 and observed using inverted fluorescent microscope, three fields were randomly chosen and the invasion capabilities of tumor cells were represented as the numbers of the invaded cells per field (scale bar: 25 μm). The experiments were repeated for three times. **(B)** Quantitative analysis of the invasive status of the cells that stably expressing shGRP78-3. The values were presented as $\bar{x}$± SE and analyzed by one-way ANOVA; (Columns,mean of three separate experiments; bars, SE; *, values significantly different at the 5% levels). **(C)** Wound healing analysis of the metastasis of the cells that stably expressing shGRP78-3. The confluent cells were wounded by sterile pipettes and the status of wound closure were observed and photographed after 24 h.the experiment was repeated for three times. (scale bar: 25 μm) **(D)** Quantitative analysis of the metastasis status of the cells that stably expressing shGRP78-3. The values were presented as $\bar{x}$± SE and analyzed by one-way ANOVA; (Columns,mean of three separate experiments; bars, SE; *, values significantly different at the 5% levels). **(E)** MTT analysis of the proliferation status of the cells that stably expressing shGRP78-3, the experiment was repeated for 3 times in tripilicate and The values were presented as $\bar{x}$± SE and analyzed by one-way ANOVA; (Columns,mean of three separate experiments; bars, SE; *, values significantly different at the 5% levels).

To explore whether GRP78 knockdown affects the invasion of HCC, we examined the invasion and motility potentialities by Transwell assay and wounding healing assay in SMMC7721 cells. Transwell assay showed that the number of invaded cells was equivalent to ~45.7% of control cells in the cells of C3 and ~34.8% in C4.These values were analyzed by one-way ANOVA and the statistical analysis revealed that these differences were significant(*p* < 0.05). These results suggested that GRP78 knockdown significantly inhibited the invasion of hepatocellular carcinoma cells(p < 0.05) (Figure 3A, B). Wound healing assay showed that the motility of C3 and C4 cells was significantly decreased as compared with control cells. The wound closure ratio was 48% for control cells, 18% for C3, and 14% for C4 respectively. The statistical analysis of one-way ANOVA revealed that the differences of these values were significant(p < 0.01) (Figure 3C, D).

In order to exclude the possibility that the inhibiton of the invasion and metastasis of GRP78 knockdown were caused by cell proliferation, we examined the proliferation statsus of C3 and C4 cells using MTT assay. Compared with control cells and parental cells, GRP78 knockdown do not affect the proliferation of SMMC7721 in 24 h, indicating that the inhibitory effect of Grp78 knockdown on the invasion and metastasis was not caused by cell proliferation (Figure 3E).

### GRP78 knockdown decreased ECM degradation

**Figure 4:**
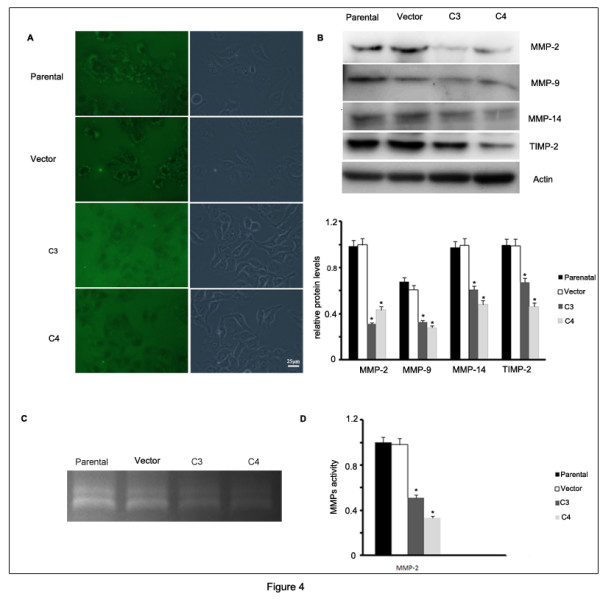
**GRP78 knockdown decreased ECM degradation. (A)** FITC-gelatin degradation analysis of the extracellular matrix degradation capability of the cells that stably expressing shGRP78-3. The experiments were repeated for three times. **(B)** Western blot analysis of MMP-2,MMP-9,MMP-14 and TIMP-2 expression in the cells that stably expressing shGRP78-3, and the results of quantative analysis were represented as $\bar{x}$± SE and analyzed by one-way ANOVA (Columns,mean of three separate experiments; bars, SE; *, values significantly different at the 5% levels). **(C)** and **(D)** Gelatin zymograph analysis of the activities of MMP-2 and MMP-9 in GRP78 knockdown cells. The activities of MMP-2 and MMP-9 were represented as $\bar{x}$± SE and analyzed by one-way ANOVA (Columns,mean of three separate experiments; bars, SE; *, values significantly different at the 5% levels).

To explore whether GRP78 knockdown influences extracellular matrix degradation, we applied FITC-gelatin degradation assay to access the matrix degradation status of parental, vector transfected, C3 and C4 cells. We observed the FITC-gelatin degradation sites which appear as visible small dots in regions under the cells in parental and vector transfected cells. However, no obvious degradation sites were seen in C3 and C4 cells, indicating that GRP78 knockdown decreased the ability of ECM degradation in SMMC7721 cells (Figure 4A). For the activity and expression of Metalloproteinase (MMPs) and tissue inhibitors of metalloproteinase (TIMPs) play critical roles in the ECM degradation [17], we detected the expression of MMP-2, 9, 14 and TIMP-2 in C3 and C4 cells by western blot. The results showed that transfection of shGRP78-3downregulated the expression levels of MMP-2, MMP-9, MMP-14 as well as TIMP-2 compared with controls. The absorptance values were analyzed using one-way ANOVA and the differences between the cells that stably expressing shGRP78-3 and control cells were significant (p < 0.05), suggesting that GRP78 knockdown decreased the expression levels of MMP-2, MMP-9, MMP-14 and TIMP-2 in SMMC7721 cells (Figure 4B and 4C). We further analyzed whether Grp78 knockdown affected the activity of MMP2 and MMP9 by gelatin-zymography assay. As shown in Figure 4D and 4E, the activity of MMP-2 in C3 and C4 cells was significantly lower than that in parental and vector transfected cells, The absorptance values were analyzed by one-way ANOVA and the differences between the cells that stably expressing shGRP78-3 and control cells were significant (p < 0.05). However, we do not detect the activity of MMP-9 in parental, vector, C3 and C4 cells. Taken together, our findings demonstrate that GRP78 knockdown inhibites the ECM degradation by decreasing the expression and activity of MMP-2.

### GRP78 knockdown decreased JNK and ERK signaling pathway

**Figure 5:**
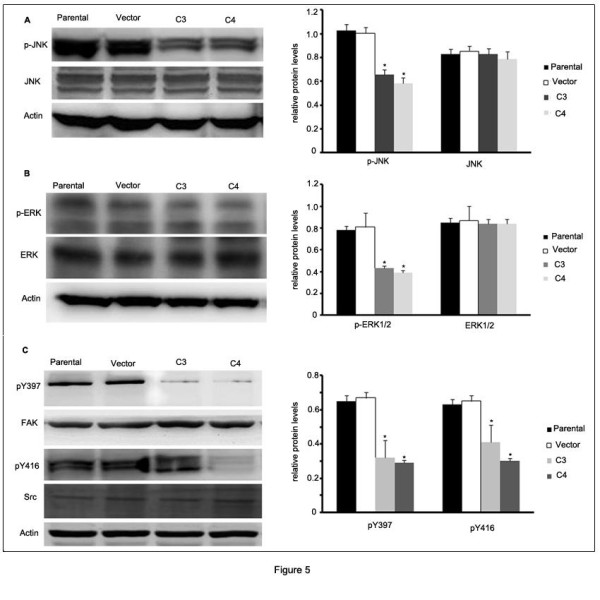
**GRP78 knockdown decreased JNK and ERK signaling pathway. (A)** Western blot analysis of JNK and p-JNK levels in cells that stably expressing shGRP78-3. **(B)** Western blot analysis of the ERK and p-ERK levels in cells that stably expressing shGRP78-3. **(C)** Western blot analysis of FAK, pY397-FAK, Src and pY416-Src in the cells that stably expressing shGRP78-3. All the experiments were repeated for three times, the results of quantative analysis were represented as $\bar{x}$± SE and analyzed by one-way ANOVA. (Columns,mean of three separate experiments; bars, SE; *, values significantly different at the 5% levels).

We then sought to determine the mechanisms underlying the reduction of MMPs activities caused by GRP78 knockdown in SMMC7721 cells. For the important roles of ERK1/2 and JNK in the regulation of MMP-2 and MMP-9 activities, we examined the phosphorylation levels of ERK1/2 and JNK in C3 and C4 cells using western blot. As shown in Figure 5A and B, the p-ERK1/2 and p-JNK levels were reduced as compared with control cells. The values were analyzed by one-way ANOVA and the differences between C3 or C4 cells and control cells were significant (*p* < 0.05). Because the activities of ERK1/2 and JNK were modulated in large part by FAK-Src signaling pathway [22], we examined the phosphorylation levels of FAK at Y397 and Src at Y416 in C3 and C4 cells. We found that GRP78 knockdown significantly decreased the levels of pY397-FAK and pY416-Src in SMMC7721 cells (*p* < 0.05) (Figure 5C). These data indicate that decreased ERK1/2 and JNK activities are involved in reducing MMP-2 activities triggered by GRP78 knockdown.

### JNK signaling is involved in the reduced MMP-2 activity caused by GRP78 Knockdown

**Figure 6:**
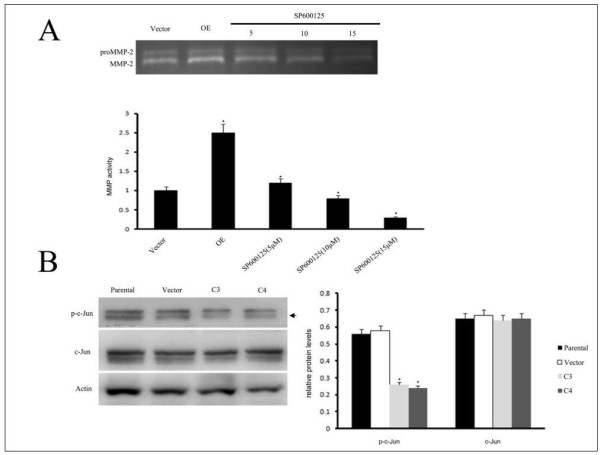
**JNK signaling is involved in the reduced MMP-2 activity caused by GRP78 Knockdown. (A)**. Gelatin zymograph analysis of MMP-2 activity in SP600125 treated GRP78 overexpressing cells (OE). GRP78 OE cells were treated with the JNK inhibitor SP600125 at various concentrations for 12 h, the conditioned medium were collected and the activity of MMP-2 were detected by gelatin zymograph. Schematic diagram of the MMP-2 activities in GRP78 OE cells that treated with SP600125, the results of quantative analysis were represented as $\bar{x}$± SE and analyzed by one-way ANOVA. (Columns, mean of three separate experiments; bars, SE; *, values significantly different at the 5% levels). **(B)** Western blot analysis of the c-Jun and p-c-Jun in the cells that stably expressing shGRP78-3 and schematic diagram of the expression levels of c-Jun and p-c-Jun. All the experiments were repeated for three times, the results of quantative analysis were represented as $\bar{x}$± SE and analyzed by one-way ANOVA. (Columns, mean of three separate experiments; bars, SE; *, values significantly different at the 5% levels).

To explore the signaling pathway involved in the reduction of MMP-2 activity induced by GRP78 knockdown, we inhibited the activity of JNK using SP600125, an inhibitor of JNK at various concentrations ranging from 5 to 15 μM in the cells that overexpressing wild type GRP78 which were established by our laboratory previously [9]. We found that the activity of MMP-2 gradually decreased with the increase of the concentration of SP600125. When the concentration rose to 15 μM, the activity of MMP-2 was almost not detected (Figure 6A and 6B). These data suggested that JNK is involved in the regulation of MMP-2 activity in GRP78 knockdown cells. To further confirm the roles of JNK in GRP78 knockdown induced reduction of MMP-2 activity, we examined the phosphorylation of c-Jun, which plays critical roles in the regulation of MMP-2 expression and activity. As shown in Figure 6C and 6D, GRP78 knockdown markedly reduced the phosphorylation level of c-Jun and the one-way ANOVA analysis revealed that the differences between C3 or C4 and control cells is significant (*p* < 0.05). Taken together; these data suggested that JNK is involved in the reduced MMP2 activity caused by GRP78 knockdown.

## Discussion

In this study, we show that knockdown of GRP78 reduces the invasiveness and metastasis in hepatocellular carcinoma cells SMMC7721, and we identify a molecular mechanism involving FAK-Src-JNK-c-Jun-MMP2 signaling pathway in these effects. These data point to a potential antitumor target for GRP78 in hepatocellular carcinoma cells.

We choose hepatocellular carcinoma cell line SMMC7721 for the establishment of in vitro invasion and metastasis model according to the expression levels of GRP78, MMP-2, MMP-9, MMP-14 and TIMP-2. We first demonstrate that knockdown of GRP78 inhibited the invasion and metastasis in SMMC7721. Many data have revealed that cell proliferation affected the outcomes of both transwell assay and wound healing assay, it is essential to examine whether GRP78 knockdown affected the proliferation of SMMC7721. In our research, we demonstrated that GRP78 knockdown do not have influence on tumor cells at the first 24 h. Taken together, these results suggested that knockdown of GRP78 decreased the invasion and metastasis of SMMC7721 and this inhibitory effect was not dependent on the proliferation of tumor cells.

Abnormal expression of MMPs is believed to play an important role in tumor cell invasion and metastasis in human cancers, including hepatocellular carcinoma [23].Among the MMPs, the roles of MMP-2 and MMP-9 in the invasiveness and metastasis of cancer cells are well characterized. In our study, we show that GRP78 knockdown reduced the expression and activity of MMP-2 in SMMC7721 cells. Although we detected MMP-9 expression by RT-PCR and western blot, we do not detect the secretion and activity of MMP-9 in SMMC7721. To elucidated this question, we examined the activities of MMP-9 in four hepatocellular carcinoma tissue samples by gelatin zymograph assay. MMP9 activities can be detected in all the four tissue samples. Since tissue samples are composed of cancer cells and surrounding non-cancer cells,which is the components of tumor microenvironment, we think that MMP-9 is secreted mainly by the non-cancer cell in tumor microenvironment.

Many data have demonstrated that MMP-14 and TIMP-2 activates pro-MMP-2 by forming a complex with TIMP-2 and pro-MMP-2. We found that GRP78 knockdown reduced the expression of MMP-14 and TIMP-2, indicating that knockdown of GRP78 decreased the expression of the members of the MMP-2 activating complex.

In this article, we further investigate the signaling mechanisms involved in the reduced MMP-2 and MMP-9 activities. Mitogen-activated protein kinases(MAPKs) are key signaling molecules controlling MMPs which is modulated large part by FAK-Src signaling pathway. We found that knockdown of GRP78 decreased the phosphorylation of JNK and ERK1/2. This is supported by our results that GRP78 knockdown downregulated the activity of FAK and Src. AP-1 complex which consists of c-Jun and c-fos plays important roles in several cellular processes. AP-1 complex translocated into the nucleus and activated the promoters containing AP-1 binding sites including the MMP-2 promoter when c-Jun was phosphorylated by JNK. Our finding that GRP78 knockdown decreased the phosphorylation of c-Jun and inhibited the translocation of AP-1 complex into nucleus. These data suggested that c-Jun was the downstream transcription factor in the reduced MMP2 activity caused by GRP78 knockdown.

Overall, our data revealed a mechanism by which GRP78 knockdown inhibits the ECM degradation and the activity and expression of MMP-2. JNK-c-Jun signaling pathway play important role in this process. This finding suggested that GRP78 may be a potential target for the prevention of the invasion and metastasis of hepatocellular carcinoma.

## Materials and methods

### Antibodies

The primary antibodies used were: GRP78 (sc-1051), GRP94 (sc-1794), MMP-2 (CST-4022), MMP-9 (CST-3852), MMP-14 (ab3644), TIMP-1 (CST-8946), TIMP-2 (sc-21735), FAK (396500, Biosource), FAK-pY397 (44625 G, Biosource), JNK (sc-7345), Src(CST-2123), Src-pY416(CST-6943), p-JNK (sc-6354), c-Jun (CST-9165), p-c-Jun (CST-9261). HRP-conjugated secondary antibodies were purchased from Zhongshan Company (Beijing, China).

### Cell culture

Human hepatocellular carcinoma cell line SMMC7721 and HepG2 were purchased from the Type Culture Collection of Chinese Academy of Science. The cells were propagated in complete DMEM medium supplemented with 10% fetal bovine serum(FBS), 2 mM glutamine, 100 U/ml penicillin, 100ug/ml streptomycin at 37°C, 5% CO_2_ -95% O_2_ and passaged every 3–5 days.

### GRP78-shRNAs transfection into SMMC-7721

The pEGFP-N1-GRP78-shRNAs were purchased from the Genechem Company (Shanghai, China). The sequences were shown as follows, all sequences were provided in 5’ → 3’ direction:

 1th: Sense: caGCATCAAGCAAGAATTGAA

 Antisense: TTCAATTCTTGCTTGATGCtg

 2th: Sense: gaCCTGGTACTGCTTGATGTA

 Antisense: TACATCAAGCAGTACCAGGtc

 3th: Sense: aaGGAGCGCATTGATACTAGA

 Antisense: TCTAGTATCAATGCGCTCCtt

 4th: Sense: aaGCAACCAAAGACGCTGGAA

 Antisense: TTCCAGCGTCTTTGGTTGCtt

Transfection was performed using Lipofectamine™ 2000(Invitrogen) as the manufacture’s instruction. Briefly, the logarithmically growing cells were plated in 6-well plate in 2000 μl of DMEM complete growth medium without antibiotics and with serum. After 24 h, 10 μl of Lipofectamine™ 2000 was diluted to 250 μl by serum-free medium, mixed with DNA solution (4 μg DNA in 250 μl serum-free medium) in a sterile 1.5 ml EP tube and incubated for 30 min at room temperature. The mixture was added drop by drop into each well, incubated for 72 h under normal cell culture conditions. pEGFP-N1 was transfected at the same time as control. The transfection efficiency was observed by fluorescent microscope and the effect of GRP78-shRNAs was determined by western blot.

### Establishment of cells that stably expressing GRP78-shRNAs

Selection of SMMC-7721 cells stably expressing GRP78-shRNAs was performed according to the manufacturer’s instructions (Invitrogen). Briefly, the complete growth medium containing GRP78-shRNAs were replaced by the selection medium containing G418 (Gibco, 400 μg/ml) after 48 h of transfection. The selection medium was replaced every 3–4 days, the clones that stably expressing GRP78-shRNAs were picked, expanded, cultured in the medium containing 200 μg/ml of G418, and identified by western blot and RT-PCR.

### RNA extraction and RT-PCR analysis

Total RNA was isolated using Trizol (Invitrogen) according to the manufacture’s recommendation. 2 μg of total RNA from each samples were reverse transcribed using oligo(dT) primers at 37°C for 90 min. The relative mRNA levels were evaluated by quantitative PCR using SYBR green PCR kit (Takara). The signals were normalized to 18 S as internal control. The primers were as follows:

 MMP-2 Forward, 5’-ATAACCTGGATGCCGTCGT-3’

 Reverse, 5’- AGGCACCCTTGAAGAAGTAGC-3’

 MMP-9 Forward, 5’-GACAGGCAGCTGGCAGAG-3’ Reverse,5’-CAGGGACAGTTGCTTCTGG-3’

 MMP-14 Forward,5’-CTGTCAGGAATGCTC-3’

 Reverse, 5’-AGGGGTCACTTGAATGCTC-3’

 TIMP-2 Forward, 5’-GAAGAGCCTGAACCACAGGT-3’

 Reverse, 5’-CGGGGAGGAGATGTAAGCAC-3’

 18 S Forward, 5’-TCAAGAACGAAAGTCGGAGG-3’

 Reverse, 5’-GGACATCTAAGGGCATCACA-3’

### Western blot-analysis

Cells were washed, harvested, lysed by lysis buffer (150 mM NaCl, 1% NP-40, 1% SDS, 1 mM PMSF, 10ug/ml Leupeptin, 1 mM Aprotinin,50 mM Tris-Cl, pH 7.4) on ice for 30 min and centrifuged at 12,000 g at 4°C for 10 min. The supernatants were quantified for protein concentration by BCA assay. Equal amounts of protein were loaded (50 μg per lane) and separated by 10% SDS-PAGE, transferred to PVDF membrane. The membrane was blocked with 5% non-fat milk for 2 h, incubated with a specific antibody (1:1000 dilution) for 3 h, stained with appropriate secondary antibody conjugated with HRP (1:2000 dilution) for 30 min at room temperature. After final washes, the membrane was developed using ECL reagent (Pierce, France). The levels of target proteins were normalized to β-Actin.

### Transwell invasion and wound healing assays

Cells were harvested and seeded onto the fibronectin-coated, porous upper chamber inserts (10^5^ per well) and allowed to invade for 48 h. After 48 h, the inserts were inverted and stained with Hochest33258. Three fields were randomly chosen and the numbers of invaded cells were counted. The invasion potentiality of the GRP78 knockdown cells was measured by the average value of penetrated cells in three fields. For wound healing assay, the monolayer was carefully wounded by sterile pipette and washed with PBS for three times to remove the debris. The wounded monolayer was cultured in DMEM containing 1% BSA for 24 h, and photographed by microscope (×100). The status of wound closure was evaluated by inverted microscope.

### Cell proliferation assay

Cells were seeded in 96-well culture plate at a density of 5 × 10^4^/ml, 100 μl each well. The status of cell viability were monitored every 24 h. Briefly, the cells were washed with PBS for 3 times, 100 μl sterilized MTT solution (0.5 mg/ml) were added into each well and the cells were incubated for 4 h in normal culture condition. After incubation, 100 μl DMSO were added to each well, and the culture plate was vortexed for 2-3 min to fully dissolve the crystallization. Finally, the absorbance at 562 nm was measured using microplate reader.

### FITC- Gelatin degradation assay

FITC-gelatin degradation assay was performed as the manufacture’s procedure (Invitrogen). In brief, coverslips (18-mm diameter) were coated with 50ug/ml poly-L-lysine for 20 min at room temperature, washed with PBS, fixed with 0.5% glutaraldehyde for 15 min and washed with PBS for 3 times. After washing, the coverslips were inverted on a drop of 0.2% FITC conjugated gelatin in PBS containing 2% sucrose, incubated for 10 min at room temperature, washed with PBS for 3 times, quenched with sodium borohydride (5 mg/ml) for 3 min and finally incubated in 2 ml of complete medium for 2 h. Cells (2 × 10^5^ each well) were plated in FITC gelatin-coated coverslips, incubated at 37°C for 12 hr. The ECM degradation status was evaluated and photographed by inverted fluorescent microscope.

### Gelatin zymography

The Conditioned medium was collected and concentrated for 2-fold by centrifugal concentrator. Equal amounts of protein were loaded and separated by 10% polyacrylamide gel containing 1 g/L gelatin. The gels were re-natured in 2.5% Triton-X-100 with gentle agitation for 30 min at room temperature. The gel was pretreated by developing buffer (5 mM CaCl2, 50 mM Tris, and 0.2 mM NaCl, 0.02% Brij35 (pH 7.5)) for 30 min at room temperature, then developed in developing buffer overnight at 37°C, stained with Coomassie Brilliant Blue R-250 for 30 minutes and destained with destaining solution. The protease activity was analyzed by gel imaging and analysis system.

### Statistical analysis

The results were represented as $\bar{x}$± SE. Difference between two experimental groups was evaluated by the students’t test and differences among groups were analyzed using One-Way ANOVA. P < 0.05 was considered to be statistically significant.

## Abbreviations

GRP78, Glucose-regulated protein 78; MMPs, Matrix metalloproteinase; ECM, Extracellular matrix; HCC, Hepatocellular carcinoma; FAK, Focal adhesion kinase; MAPK, Mitogen-activated protein kinase; ERK, Extracellular-regulated kinases; JNK, c-Jun N-terminal kinases.

## Competing interests

We have no financial or other conflicts of interest that might influence the results or interpretation of our study.

## Authors’ contributions

Conceived and designed the experiments: Rongjian Su, Junsheng Luo. Performed the experiments: Hongdan Li, Huijuan Song, Jia Liang and Song Zhao. Analyzed the data: Hongdan Li and Huijuan Song. All authors read and approved the final manuscript.
